# Ridesharing as an Alternative to Ambulance Transport for Voluntary Psychiatric Patients in the Emergency Department

**DOI:** 10.5811/westjem.2020.2.45526

**Published:** 2020-04-21

**Authors:** Andrea Blome, Jennifer Rosenbaum, Nicole Lucas, Kraftin Schreyer

**Affiliations:** Temple University Hospital, Department of Emergency Medicine, Philadelphia, Pennsylvania

## Abstract

**Introduction:**

Emergency department (ED) crowding is a growing problem. Psychiatric patients have long ED lengths of stay awaiting placement and transportation to a psychiatric facility after disposition.

**Methods:**

Retrospective analysis of length of ED stay after disposition for voluntary psychiatric patients before and after the use of Lyft ridesharing services for inter-facility transport.

**Results:**

Using Lyft transport to an outside crisis center shortens time to discharge both statistically and clinically from 113 minutes to 91 minutes (p = 0.028) for voluntary psychiatric patients. Discharge time also decreased for involuntary patients from 146 minutes to 127 minutes (p = 0.0053).

**Conclusion:**

Ridesharing services may be a useful alternative to medical transportation for voluntary psychiatric patients.

## INTRODUCTION

Emergency department (ED) crowding threatens patient safety and public health.^1^ Crowding exists when there is a lack of space or resources in an ED required to meet the timely needs of the next patient.^2^ Such an environment leads to medical errors and inferior care.^3^,^4^ One study demonstrated that ED crowding is associated with increased mortality in patients awaiting transfer to an intensive care unit.^5^ The issue of ED crowding is growing on a national scale, with the Centers for Disease Control and Prevention estimating that 50% of EDs experience crowding and 45% of United States hospitals have been on ambulance diversion sometime in the previous year.^6^,^7^ Consequently, EDs are focusing on finding solutions. Efforts such as performing registration at the bedside and placing a physician in triage have been shown to improve ED efficiency.^8^,^9^ Despite an understanding that patients requiring transportation to their discharge destination have longer discharge times, little research has been performed looking at solutions to this crucial step in eliminating ED crowding.^10^,^11^

Patients, especially the elderly, disabled, or economically disadvantaged, may experience difficulty obtaining transportation home.^12^ Psychiatric patients, in particular, are also known to have lengthy disposition times while awaiting placement and transportation to a psychiatric facility. One study suggested psychiatric patients spent an average of 11 hours in the ED when seeking care.^13^ There are many barriers that contribute to this long length of stay (LOS) including insurance status/type, day of presentation to the ED, hand-offs, Emergency Medical Treatment and Labor Act paperwork, patient behavior/use of medications, and delays in medical transportation.^14^,^15^ This transportation barrier has been shown to have many negative downstream effects for other patients, including delays in diagnosis and treatment, as well as contributing to ED crowding.^14^

The advent and broad availability of ridesharing services such as Lyft and Uber may be of use to patients with limited access to transportation. One study estimated that 85% of patients were aware of ridesharing services, and of that percentage, 5% planned to use this transportation method upon discharge.^15^ Ridesharing services may be a great option for older adults who are unable to drive or patients with limited financial means. However, studies have shown that elderly patients and patients with less income and education have limited knowledge and utilization of these services.^15^,^16^ These ridesharing services have also piqued the interest of hospital systems aiming to provide non-emergency medical transportation services to their patients.^17^–^19^ These efforts are bolstered by studies in primary care demonstrating improved show-rates when using ridesharing services, although other studies have not definitively demonstrated benefits in decreasing no-show rates.^22^,^23^

Despite the broad availability of ridesharing services and their increasing utilization by hospital systems, there are no studies on the use of taxi services and limited research on the effect of ridesharing on ED LOS after discharge or interfacility transport. This study aims to determine the role of ridesharing services on ED LOS for patients awaiting voluntary transportation to a psychiatric facility.

## METHODS

We performed a retrospective analysis of time from disposition until a patient’s respective discharge on a cohort of patients requiring transportation to psychiatric services before and after a hospital ridesharing initiative was implemented. The study was performed during an eight-month period between December 1, 2018–July 29, 2019, at an urban, university-associated ED and Level I trauma center serving an average annual patient population of approximately 85,000 in Philadelphia, Pennsylvania. The chart abstractors were not blinded to the study hypothesis. The study coincided with the advent of an ED initiative of organizing and paying for ridesharing services using Lyft for discharged patients requesting voluntary transfer to a psychiatric facility. This service was provided to all patients requesting to go to a psychiatric facility on a voluntary basis, as well as those requesting detox or drug rehabilitation.

At the study ED, patients presenting with psychiatric concerns are evaluated and determined to require either involuntary evaluation and treatment or voluntary transfer to a psychiatric facility. Any patient 14 years of age or older who is experiencing a mental health crisis and wishes to seek inpatient care for their safety may request voluntary commitment. The ED is affiliated with a local crisis response center (CRC) that is a 24-hour psychiatric emergency service where patients are seen as walk-ins and includes a 23-hour observation unit that can admit to an inpatient behavioral health center.

Before the introduction of the ridesharing initiative, all patients were provided medical transportation, in either an ambulance van or wheelchair van by a contracted medical transportation company to the CRC regardless of their voluntary or involuntary status. After the initiative, all patients requesting psychiatric services voluntarily, detox or drug rehabilitation, were eligible for a free Lyft transport to the CRC. Medical transportation was still obtained for all patients who were determined to require involuntary evaluation.

All patients transferred from the ED to the CRC were reviewed during this eight-month study period. Using R statistical programming software, we used two sample t-tests to evaluate the effect of using Lyft transport on the mean discharge time in minutes, for all eligible and ineligible subjects. In addition, we collected data to determine whether patients transferred to the CRC were evaluated by a psychiatrist or left prior to evaluation. A chi-squared test was completed to assess whether there was a statistically significant change in the number of patients who completed a psychiatric evaluation at the CRC before and after the Lyft transport protocols were commenced.

## RESULTS

We included 814 patients in this study. Patients eligible for Lyft transport included those who voluntarily committed to a psychiatric evaluation at the CRC, as well as those who requested transport for drug rehabilitation or detox. Ineligible patients were those who were involuntarily sent for a CRC evaluation. A total of 410 patients were included prior to the initiation of the Lyft initiative, from December 1, 2018 until March 21, 2019. Of these patients 242 were ineligible and 168 were eligible for Lyft transport. In addition, 404 patients were included after the Lyft initiative commencement, from March 21–June 29, 2019. There were 286 patients who were not eligible for Lyft and 118 who were eligible.

We used two sample t-tests to evaluate the effect of Lyft transport on patient discharge time in minutes. Eligible patients, n = 286, saw a statistically significant drop in mean minutes to discharge, decreasing from 113 minutes to 91 minutes (p = 0.028). The ineligible patients, n = 582, also saw a statistically significant reduction in discharge times. When Lyft was used for other eligible patients, the mean discharge time for ineligible patients decreased from 146 minutes before the initiation of Lyft to 127 minutes during the Lyft initiative (p = 0.0053) (Table 1). We used a chi-square test to determine whether there was a significant difference between the number of patients seen in the CRC before and after the initiation of Lyft. Prior to the Lyft initiative, 90.5% of eligible patients were seen by psychiatrists at the CRC after ambulance transport. After commencement of the initiative, 83.9% of eligible patients completed a psychiatric evaluation at the CRC. Per chi-squared testing, this change in value was not statistically significant (p = 0.0947) (Table 2).

## DISCUSSION

This study showed that using Lyft transport to transfer voluntary psychiatric patients to a crisis center decreases the discharge times after disposition of both voluntary and involuntary psychiatric patients. These findings are both statistically and clinically significant. ED psychiatric patients are known to experience lengthy time to discharge due to the lack of available inpatient psychiatric beds. For those patients who simply require transportation to a psychiatric facility and have already been medically cleared by an emergency physician, ridesharing serves as an adequate alternative to traditional medical transportation.

Ridesharing is an efficient alternative and has the potential for significant cost savings for any hospital and patient, as these services cost less than any mode of medical transportation. This more efficient discharge process is also specifically important for patients with substance use disorder or mental illness. The process streamlines obtaining help for drug or alcohol addiction in patients at risk for experiencing withdrawal symptoms while waiting. There was also no statistically significant change in the number of patients who ultimately received a CRC psychiatric evaluation, indicating that the change in transportation mode likely did not influence the chance that a patient would be evaluated in the CRC.

In addition, the use of Lyft vehicles reduces the utilization of ambulance transport services, which in turn increases availability for other patient transports. In our study, ineligible patients (ie, those under involuntary status) also showed a clinically and statistically significant decreased transport time by about 20 minutes. Lastly, the reduced turnaround times for psychiatric patients frees additional treatment space for other patients waiting to be seen.

## LIMITATIONS

One limitation of the study was a potential lack of generalizability to non-psychiatric patients. In addition, the chart abstractors were not blinded to the study hypothesis. Nor did the study address the issue of patients boarding due to unavailability of an inpatient bed. Furthermore, although there was a non-statistically significant 7% decrease in the number of patients who were ultimately evaluated by a psychiatrist in the CRC in those patients using a Lyft, we were unable to determine the reason(s) for not obtaining a psychiatric evaluation. Therefore, it is unlikely, but unknown whether this decrease was a direct result of using Lyft transportation. CRC-specific information, including CRC wait times and CRC boarding times for inpatient beds were not analyzed in this study. It is unknown whether expedited transfers objectively decreased a patient’s wait time to their final disposition from the CRC. Lastly, other potential confounders were not analyzed, such as the effect of volume on the data.

## CONCLUSION

Patients seeking or requiring psychiatric services from the ED often have lengthy wait times. This retrospective analysis sought to identify whether using a ridesharing service decreased length of stay for psychiatric patients who are seeking voluntary psychiatric evaluations. Using Lyft decreased the time that patient’s waited for transportation after disposition from the ED with both clinical and statistical significance. As a result, the time that voluntary psychiatric patients spent waiting for transportation was reduced, with an additional significant reduction in times for involuntary patients. Ridesharing is a viable, cost-effective option for psychiatric patients seeking voluntary treatment and for whom transportation is the only barrier.

## Figures and Tables

**Table 1:** Pre- and Post-Lyft Comparison of Length-of-Stay for Eligible and Ineligible Patients.

Patient group	Number of patients		Mean length of stay after disposition (minutes)		Mean change in length of stay after disposition (μ1–μ2)	P-value: μ1 > μ2

	Pre-Lyft	Post-Lyft	Pre-Lyft (μ1)	Post-Lyft (μ2)		
						
Ineligible	242	286	146.3	126.5	19.75 95% CI: (5.901, 33.59)	0.0053
Eligible	168	118	112.8	91.38	21.41 95% CI: (2.364, 40.45)	0.0278

*CI*, confidence interval.

**Table 2:** Comparison of Eligible Patients Using Lyft and Completion of Respective Psychiatric Evaluations.

	Completed psychiatric evaluation	No psychiatric evaluation
Pre-Lyft	152	16
Post-Lyft	99	19

Chi-square statistic: p = 0.095
